# A simple and green capillary electrophoresis-mass spectrometry method for therapeutic drug monitoring of colistin in clinical plasma samples

**DOI:** 10.1016/j.heliyon.2023.e23111

**Published:** 2023-11-30

**Authors:** Ivana Cizmarova, Vojtech Parrak, Peter Secnik jr, Peter Secnik, Ladislav Sopko, Kristina Nemergutova, Andrej Kovac, Peter Mikus, Juraj Piestansky

**Affiliations:** a Department of Pharmaceutical Analysis and Nuclear Pharmacy, Faculty of Pharmacy, Comenius University in Bratislava, Odbojarov 10, SK-832 32 Bratislava, Slovakia; b Toxicological and Antidoping Center, Faculty of Pharmacy, Comenius University in Bratislava, Odbojarov 10, SK-832 32 Bratislava, Slovakia; c Institute of Neuroimmunology, Slovak Academy of Sciences, Dubravska cesta 9, 84510 Bratislava, Slovakia; d SK-Lab s.r.o., Partizanska 15, SK-984 01, Lucenec, Slovakia; e Clinic of Hematology and Transfusiology, St Cyril and Methodius Hospital, Antolska 11, SK-851 07, Bratislava, Slovakia; f Department of Galenic Pharmacy, Faculty of Pharmacy, Comenius University in Bratislava, Odbojarov 10, SK-832 32 Bratislava, Slovakia

**Keywords:** Capillary electrophoresis, Mass spectrometry, Colistin, Therapeutic drug monitoring, Clinical samples

## Abstract

Colistin and other polymyxin antibiotics have become increasingly used in clinical settings as a result of treating multidrug-resistant infections in critically ill patients. The highly variable pharmacokinetics of colistin in these patients is accompanied by a high risk of toxicity or underdosing. An effective tool that allows rational optimization of the drug dosage regimen is therapeutic drug monitoring. Therefore, there is a need to dispose with appropriate, sensitive, and accurate analytical methods. Here, a simple, specific, and accurate on-line capillary electrophoresis – tandem mass spectrometry method was developed and applied for the first time to determine colistin in human plasma. Protein precipitation using acidified acetonitrile was the solitary procedure used to achieve sample pretreatment. A bare fused silica capillary was employed for the separation process, and the background electrolyte used was 50 mM formic acid (pH 2.54). The FDA's bioanalytical method validation guidelines were followed in the validation of the proposed method. For colistin A and colistin B, favorable performance and validation parameters were obtained (such as linearity, limit of detection, lower limit of quantitation, intra-day and inter-day precision, accuracy, and stability).The validated method was then effectively used to analyze real clinical samples taken from patients who were in critical condition. Our newly developed method is comparable with previously published liquid chromatography approaches and has the potential to be applied in the therapeutic monitoring of colistin in routine clinical laboratories. Moreover, according to the greenness assessment, the developed capillary electrophoresis – mass spectrometry method represents a very interesting green and sustainable tool in the field of bioanalysis.

## Highlights

• The first CZE-MS/MS approach for the determination of colistin in human plasma.
• LOD at ng.mL^−1^ concentration level.
• Simple sample preparation and low plasma sample consumption (only 30 μL).
• Therapeutic drug monitoring of critically ill patients.
• Green analytical chemistry method.

## Graphical abstract

**Figure:**
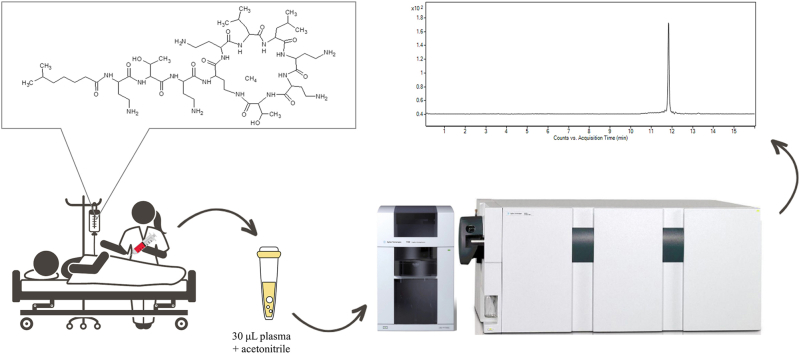

## 1 Introduction

Colistin (CST; or polymyxin E) is an antibiotic (ATB) that was isolated from a strain of *Bacillus polymyxa* var. *colistinus* in 1947 [1]. Its use in clinical practice was abandoned in the 1970s because of the high risk of neurotoxicity and nephrotoxicity [2]. CST, which is currently employed as a last-line ATB for the treatment of infections caused by gram-negative bacteria, has reemerged as a result of a rise in drug-resistant bacterial species that are both widespread and multi-drug resistant (such as *Pseudomonas aeruginosa*, *Acinetobacter baumannii*, and *Klebsiella pneumoniae*) [3]. Because of the still-growing bacteria resistance to ATB treatment, the therapeutic strategies are based on effective doses and concomitant administration of ATBs [[4], [5], [6]]. Both of these strategies are typically supported by therapeutic drug monitoring (TDM).

**Fig. 1:**
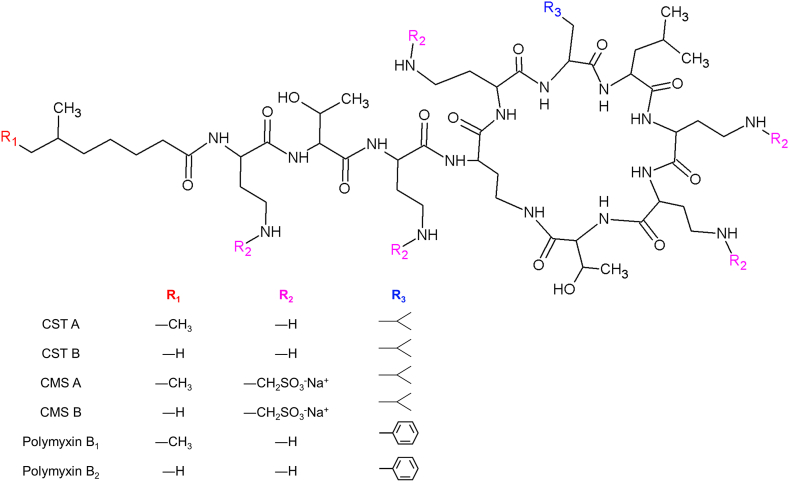
Chemical structure of CST A, CST B, colistin methanesulfonate A (CMS A), colistin methanesulfonate B (CMS B), polymyxin B_1_ and polymyxin B_2_.

Chemically, CST is a decapeptide formed by a heptapeptide ring with an attached tripeptide side chain linked to a fatty acyl chain (Fig. 1) [7]. Commercially available CST products are mixtures of 36 distinct lipopeptides [8], and only 11 of them have their chemical structure elucidated [9]. The main components are colistin A (CST A) and colistin B (CST B), which differ from each other by only one methylene group in the fatty chain residue (CST A contains 6-methyl octanoic acid and CST B contains 6-methyl heptanoic acid) [10,11].

On the drug market, CST is available in two forms: colistin sulfate, which is used topically and orally (its absorption from the gastrointestinal tract is minimal), and a less toxic and inactive prodrug for parenteral and inhalation therapy – colistin methanesulfonate (CMS) [12]. A sulfomethyl group is added to CST's primary amines to create CMS, a chemically modified version of CST. Consequently, hydrolysis of CMS in aqueous solutions is associated with the creation of 32 different products, including CST [3,13]. According to the European Pharmacopoeia, the required content of CST in drugs on the market is expressed as the sum of two dominant components, i.e., CST A and CST B, and three minor components (polymyxin E3, polymyxin E1-7MOA (7-methyl octanoyl), polymyxin E1-I) which should not be less than 77 %. Furthermore, the maximum content of each of the three minor components mentioned above should be <10 % [8,14]. Since CST came on the market before strict criteria for marketed drugs were established, no trials are designed to test the resulting product [15,16]. However the ratio of CST forms differs across manufacturers as well as between batches produced by the same manufacturer. The variability in CMS products affects the formation of antimicrobial active CST [3]. Moreover, there is also a difference in the binding of the two CST forms (CST A – 56.6 %; CST B – 41.7 %) to plasma proteins (especially to acidic alpha glycoprotein) [11], resulting in changes in the concentration of the unbound fractions of each component [17]. It was demonstrated that commercially available CST products have lower *in vivo* efficacy than both their respective components CST A and CST B, suggesting that minor components of lipopeptides are less effective *in vivo* [9,11]. Although CMS has been used in critically ill patients for a long time, its pharmacokinetics (PK) is still not well understood, and optimal dosing strategies are being sought [18]. The patient's renal function should determine the CMS dosage [19], but it is difficult to predict plasma CST concentrations in individual patients [20]. Therefore, TDM is highly recommended owing to CST variable PK and the narrow therapeutic window. Optimizing CST dosage in accordance with observed plasma concentrations can greatly enhance antibacterial efficacy while reducing the development of toxicity and resistance [21]. The total sum of CST A and CST B is used to quantify plasma CST concentration for TDM purposes [12]. It is recommended to determine the free concentration of CST because this concentration is responsible for antibacterial activity [22]. On the other hand, the overall CST concentration is often measured. This is because it is only strongly advised to monitor the free drug fraction for drugs that have a binding to plasma proteins of more than 80 % [23]. In the literature published to date, the determination of CST in biological samples was performed by high-performance liquid chromatography (HPLC) coupled with fluorescence or mass spectrometry (MS) detection [[24], [25], [26], [27], [28], [29], [30], [31], [32], [33], [34], [35], [36], [37], [38], [39], [40], [41], [42], [43], [44], [45], [46]]. These methods were relatively fast (from 2 to 20 min), required various sample pretreatment – typically protein precipitation (acetonitrile, trifluoroacetic acid) and/or solid phase extraction (SPE), and were characterized by the lower limit of quantitation (LLOQ) or limit of quantitation (LOQ) values ranging from 2 to 500 ng mL^−1^.

Chromatographic methods represent the gold standard in clinical and biochemical laboratories, but there is also a growing interest in electrophoretic methods (especially capillary zone electrophoresis – CZE). It is a consequence of their high separation efficiency, fast analytical response time, simplicity of performance, ecological aspect (separation performed in aqueous media), and notable cost efficiency [[47], [48], [49], [50], [51], [52]]. Despite these advantages, only a few papers deal with CST analysis by capillary electrophoresis (CE). A CZE-UV approach was used for the determination of CST A and CST B in pharmaceutical products [53]; an in-house flow-injection CE method with capacitively coupled contactless conductivity (C^4^D) detection was developed for the direct measurement of CST in pharmaceutical and human serum samples [54]; and a micellar electrokinetic chromatography (MEKC) method was applied to separate various polymyxins, including CST [55]. While on-line CE-MS hyphenation is a sophisticated, modern technique that may separate analytes based on variations in their electrophoretic mobilities and subsequently provide information about the molecular masses of substances that have been analyzed [56], no work has yet been published applying this approach to the analysis of CST in plasma or serum. Therefore, our recent work deals with the development of a simple, rapid, sensitive, and accurate CE-MS method for the determination of CST in plasma samples. The simplicity of our pioneering approach is supported by the biological sample pretreatment based only on protein precipitation with the use of acetonitrile. The method was developed with an emphasis on its future application in clinical practice for the possibility of providing routine TDM of CST in critically ill patients.

## 2 Materials and methods

### 2.1 Chemicals and samples

Merck (Darmstadt, Germany), Sigma Aldrich (Steinheim, Germany), and Fluka (Buchs, Switzerland) provided the LC-MS grade chemicals required to prepare the electrolyte solutions (formic acid – HFo, acetic acid – HAc), as well as the sheath liquid (methanol – MeOH, isopropyl alcohol – IP, and ammonium acetate – NH_4_Ac). LC-MS grade acetonitrile (ACN) was obtained from VWR International (Vienna, Austria). Sodium hydroxide (NaOH), p.a. quality, was obtained from Agilent Technologies (Santa Clara, CA, USA). The electrolytes, sheath liquid, and samples were prepared using demineralized water, which was produced using a Millipore Simplicity 185 (UV) water purification system (Millipore, Molsheim, France). Electrolytes were kept in the refrigerator before analysis and filtered using disposable membrane filters with a 0.22 μm pore size from Millipore.The colistin sulfate analytical-grade reference standard and polymyxin B sulfate analytical-grade standard were purchased from Sigma Aldrich (Steinheim, Germany).

### 2.2 Instrumentation

All CE-MS experiments were performed using an Agilent 7100 capillary electrophoresis system (Agilent Technologies, Santa Clara, CA), coupled with an Agilent 6410 Series Triple Quadrupole tandem mass spectrometer (Agilent Technologies) with a commercial coaxial sheath liquid electrospray (ESI) interface. Separation was realized in a 99 cm × 50 μm ID bare fused silica capillary (MicroSolv Technology Corporation, Eatontown, NJ). The samples were injected hydrodynamically for 20 s at 50 mbar. Following the sample, the separation system was hydrodynamically injected with a brief zone of background electrolyte (BGE) under 2 s of 50 mbar pressure. This step enhances the sample's quantitative injection and reproducibility. The experiments were carried out with a voltage of +25 kV and normal polarity, yielding currents of 5–8 μA. Furthermore, an internal pressure of 5 mbar was applied from the second minute of the electrophoretic analysis. The sheath liquid composed of MeOH and 0.1 % HFo water solution (50/50, v/v) was delivered by an Agilent 1260 Infinity isocratic LC pump (Agilent Technologies) at a flow rate of 8 μL min^−1^. The MS was operated in positive ion, multiple reaction monitoring (MRM) mode using characteristic precursor ion – product ion mass transitions for each substance. The dwell time (the period during which MS collects data for a certain MRM transition) was 150 ms. Further MS parameters were set as follows: capillary voltage – 5000 V, nebulizing gas (nitrogen) pressure – 5 psi, drying gas (nitrogen) temperature – 300 °C, and drying gas (nitrogen) flow – 5 L min^−1^.

### 2.3 Capillary treatment

A new separation capillary was activated and conditioned before to its first use by flushing it with an aqueous solution of 1 M NaOH for 15 min. After that, the capillary was preconditioned with BGE for 10 min and rinsed with demineralized water for 15 min. These actions were all carried out at 950 mbar of pressure. Before each injection phase, the separation capillary was flushed with BGE for 2 min and a negative voltage of −25 kV was applied for 30 s to achieve re-equilibration. MeOH was used to cleanse the capillary for 2 min following each run. Preconditioning and postconditioning reduced carry-over and improved analysis repeatability. Each day's end involved rinsing the capillary for 20 min with demineralized water, 10 min with BGE, and an overnight stay in BGE. Every step was carried out at a steady laboratory temperature.

### 2.4 Preparation of the sample and standard solution

1 mg of the powder was dissolved in 1 mL of demineralized water to prepare the stock solutions for the polymyxin B internal standard (IS) and the CST reference standard. Aliquoted stock solutions (200 μL) were kept for a maximum of four days at −20 °C.

Every day, adequate dilution of the stock solution with demineralized water was used to prepare working solutions of CST sulfate. The IS working solution was prepared daily by mixing 7 μL of IS stock solution, 10 μL of HFo, and 9983 μL of ACN. Based on six consecutive measurements, the CST A and CST B ratio in the standard was found. When adjusting the sum of the area of both substances to 100 %, the amount of CST A was 37.2 % and the amount of CST B was 62.8 %. The acquired results showed good consistency with the information reported in other articles [32,37,39]. This information was used to calculate the concentrations of both analytes present in the CST sulfate standard solutions prepared in the range 0.5–20 μg mL^−1^ (individual standard concentration: 0.5, 1, 5, 10, 15 and 20 μg mL^−1^). The individual standard concentrations were equivalent to 0.19, 0.37, 1.86, 3.72, 5.58, 7.44 μg mL^−1^ of CST A sulfate and 0.31, 0.63, 3.14, 6.28, 9.42, 12.56 μg mL^−1^ of CST B sulfate in the injected calibration solutions prepared in water and plasma samples (pooled plasma from six healthy volunteers).

Quality control (QC) samples were prepared at three concentration levels: i.) low QC: 0.28 μg mL^−1^ (CST A sulfate) and 0.47 μg mL^−1^ (CST B sulfate); ii.) medium QC: 2.79 μg mL^−1^ (CST A sulfate) and 4.71 μg mL^−1^ (CST B sulfate); and iii.) high QC: 6.51 μg mL^−1^ (CST A sulfate) and 10.99 μg mL^−1^ (CST B sulfate). The calibration curve and the QC sample solutions were prepared by mixing 30 μL of the CST sulfate standard at the desired concentration level with 30 μL of demineralized water or plasma and 120 μL of the IS working solution. After being vortexed and left to rest at room temperature for 10 min, the samples were centrifuged at 30,000×*g* for 10 min. Following this action, supernatant was immediately injected from a CE vial. Every sample underwent three measurements.

Clinical plasma samples from two critically ill patients were obtained from the Clinic of Hematology and Transfusiology of St Cyril and Methodius Hospital in Bratislava. After collection, the samples were stored at −80 °C. Before the analysis, the samples were thawed at laboratory temperature, vortexed, and then, 30 μL of the plasma sample was mixed with 30 μL of demineralized water and 120 μL of the IS working solution. The samples were centrifuged at 30,000×*g* for 10 min after being kept at room temperature for 10 min. Following this action, the supernatant was injected directly into the CE system from a CE vial.

## 3 Results and discussion

### 3.1 Optimization of the CE separation conditions

**Fig. 2:**
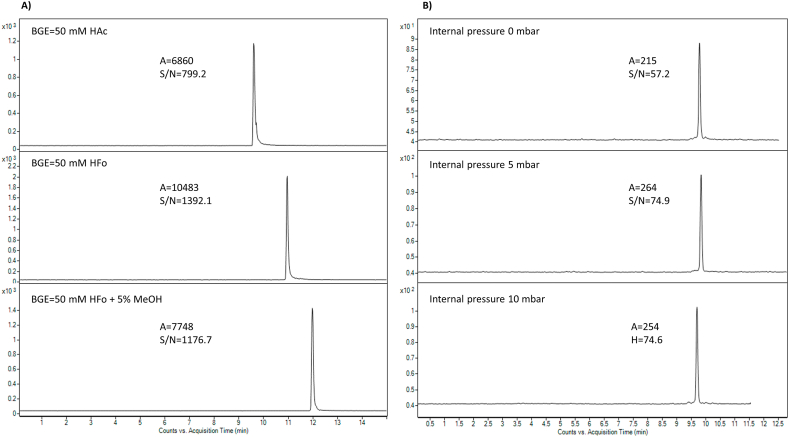
A) Optimization of the BGE composition. Multiple reaction monitoring (MRM) profiles of CST B (*m*/*z* transition 386.0 → 101.0) were obtained under various separation conditions – i.e. 50 mM HAc, 50 mM HFo, and 50 mM HFo with the addition of 5 % MeOH. The concentration of injected CST sulfate was 100 μg mL^−1^, which represented a concentration 62.8 μg mL^−1^ of CST B sulfate. The sample was injected hydrodynamically using a pressure of 50 mbar for 20 s. B) Optimization of the internal pressure (0–10 mbar) during CE analysis and its effect on the CST analytical signal intensity. The CZE profiles were obtained with the use of the quantitative MRM *m*/*z* transition of CST B, i.e. 386.0 → 101.0. The optimization was performed on a plasma sample spiked with the CST sulfate at a concentration level of 5 μg mL^−1^, which corresponds to the CST B sulfate concentration of 3.14 μg mL^−1^. The sample was injected hydrodynamically using a pressure of 50 mbar for 20 s. The applied voltage was 25 kV and 50 mM HFo was used as BGE. For further details see section 2.2.

Colistin is a polycationic peptide that exhibits basic properties due to the presence of five free amino groups of diaminobutyric acids in its structure [57]. Since acidic conditions are typically seen during the analysis of therapeutic peptides by CE-MS [58], aqueous solutions of HFo and HAc were evaluated as appropriate BGE. These BGEs fulfill the required criteria on the CE-MS connection – i.e., volatility and low ion strength. The tested electrolyte concentration range was 20–500 mM (20, 50, 100, 200, and 500). Fig. 2A clearly shows that the use of HFo as BGE was accompanied by a higher analytical signal and a better signal-to-noise (S/N) ratio for CST. Therefore, BGEs composed of HFo were investigated in more detail. Table 1 summarizes how HFo concentration affects the CST analytical signal's stability and intensity. The increase in BGE concentration was associated with a decrease in the peak area of CST at a minimum at the concentration of 100 mM. Further increase in BGE concentration led to a slight increase in peak area. A similar behavior was observed for the CST peak intensity. According to the obtained data, it can be stated that the most suitable analytical conditions represented by appropriate peak area, peak intensity, and their reproducibility (expressed as a relative standard deviation – RSD, of three measurements) were obtained with the use of 50 mM HFo. Additionally, the effect of organic modifiers as BGE additives on the separation behavior of CST was investigated. The separation and detection properties were not improved by adding 5 % MeOH to BGE, as Fig. 2A illustrates. The use of organic modifiers was accompanied by a decrease in peak area and S/N ratio, and their relatively high RSD values (12.3 %, 9.9 %), and prolongation of the migration time.

**Table 1:** Optimization of the background electrolyte (BGE) composition of the CE-MS method for CST determination measured at 100 μg mL^−1^ concentration level.

BGE	pH	Area normalized (%)	RSD area (%), n = 3	Height normalized (%)	RSD height (%), n = 3
HFo (mM)					
20	2.75	100	6.5	94.7	6.0
50	2.54	97.7	2.5	100	0.4
100	2.39	76.4	6.8	69.8	11.2
200	2.23	85.0	6.1	82.5	7.5
500	2.03	87.2	11.6	87.3	54.3

The adsorption of peptide and protein samples on the capillary wall frequently affects their analysis. The best way to get around this problem is to employ a low-pH electrolyte that can reduce the electroosmotic flow (EOF) and suppress silanol group dissociation. Moreover, the concurrent application of positive pressure at the capillary inlet can shorten the separation time and increase ESI stability [59]. Therefore, the effect of the application of internal pressure (0, 5, and 10 mbar) during separation on the analytical signal was investigated (Fig. 2B). The introduction of an internal pressure of 5 mbar from the second minute of the analysis led to a more than 20 % increase in the analytical signal of the CST compared to the analysis without applied internal pressure. An additional increase in internal pressure to the value of 10 mbar did not show no additional benefit. Therefore, the internal pressure of 5 mbar was used for all subsequent measurements.

### 3.2 Optimization of MS detection conditions

A two-stage optimization of the MS conditions was realized. This procedure covered the investigation of ESI and MS analyzer parameters.

#### 3.2.1 ESI optimization

Most of the MS approaches used for the analysis of CST were performed in positive ESI mode [12], but some of them were also done in negative mode [29,32]. We used the positive ESI mode because of the positive charge of the analyte in the separation environment.

**Fig. 3:**
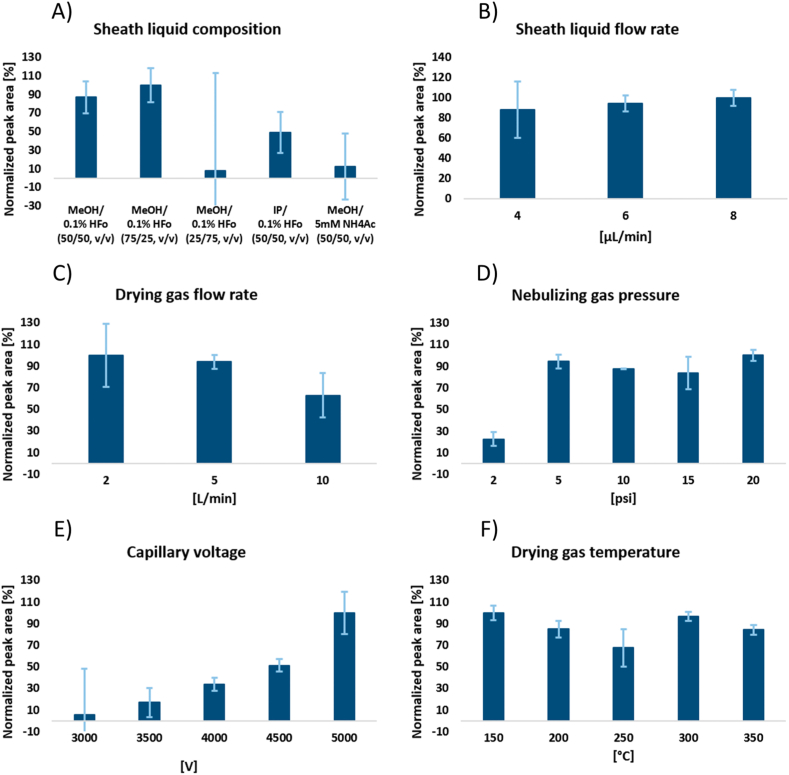
Optimization of the ESI-MS working conditions: A) sheath liquid composition, B) sheath liquid flow rate, C) drying gas flow rate, D) nebulizing gas pressure, E) capillary voltage, F) drying gas temperature. The concentration of the analyte – CST sulfate was 10 μg mL^−1^. The sample was injected hydrodynamically using a pressure of 50 mbar for 20 s. The applied voltage was 25 kV and 50 mM HFo was used as BGE.

The sheath liquid is a crucial factor of the coaxial sheath flow ESI interface because it ensures adequate ionization of the analyte and provides electrical contact between the liquid in the separation capillary and the electrode [60]. In order to prepare sheath liquid, organic solvents and water are typically combined with volatile organic acids or bases. Three different kinds of sheath liquid combinations were examined here.: a) MeOH/0.1 % HFo water solution (50/50, v/v), (75/25, v/v), (25/75, v/v); b) IP/0.1 % HFo water solution (50/50, v/v); and c) MeOH/5 mM NH_4_Ac water solution (50/50, v/v). In order to achieve the highest possible analytical signal and ensure signal reproducibility (shown by the relative standard deviation of the peak area, RSD_area_), optimization was done. At first, various mixtures at 50/50 (v/v) were tested and compared. Best outcomes were attained, when the mixture of MeOH/0.1 % HFo water solution was used. Compared to other sheath liquid mixtures, under such composition, the highest intensity and reproducibility of the CST analytical signal was obtained (Fig. 3A).

Furthermore, different MeOH and 0.1 % HFo aqueous solution ratios were investigated. The 25/75 (v/v) ratio generated extremely low signal intensity and high RSD values. The MeOH/0.1 % HFo water solution mixture (75/25, v/v) showed a slightly higher signal intensity compared to the initial mixture (50/50, v/v). However, a significant decrease in the S/N ratio was observed (Fig. S1 in Supporting Information). Finally, the mixture of MeOH and 0.1 % HFo water solution (50/50, v/v) was selected as the optimal sheath liquid.

Similarly, the sheath liquid flow rate is important since it influences the stability and sensitivity of MS detection as well as the efficacy of the ionization process. In this case, 4–8 μL min^−1^ of sheath liquid flow rate were examined. Based on the acquired data, the best sheath liquid flow rate was determined to be 8 μL min^−1^, which also included a steady ESI and analytical signal. This resulted in the greatest S/N ratio (Fig. 3B).

Additional ESI parameters were investigated and optimized in the following ranges: nebulizing gas pressure (2–20 psi), drying gas temperature (150–350 °C), drying gas flow rate (2–10 L min^−1^), and capillary voltage (3000–5000 V). These parameters are crucial for a successful ionization process and stability of the analytical signal. Fig. 3C–F provide a summary of the outcomes of the optimization process for the previously discussed parameters., and the following values of the investigated parameters were used in the next measurements: nebulizing gas pressure 5 psi, drying gas temperature 300 °C, drying gas flow rate 5 L min^−1^ and capillary voltage 5000 V.

#### 3.2.2 Triple quadrupole (QqQ) MS optimization

**Fig. 4:**
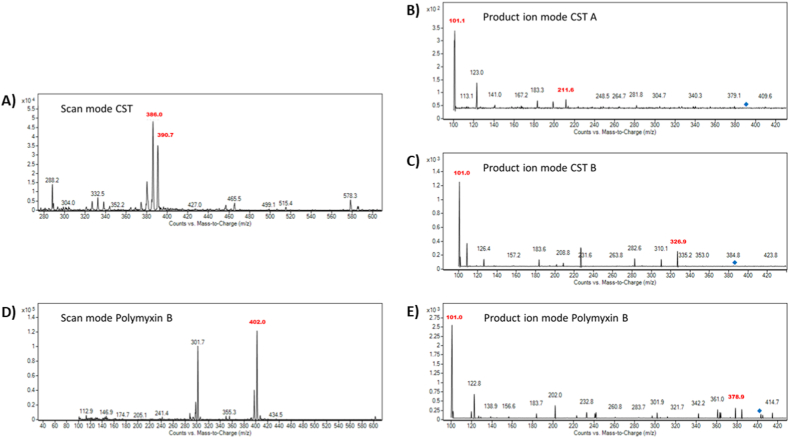
Mass spectra of parent and product ions of CST A, CST B, and polymyxin B. The spectral profiles were used for the selection of product ions serving for the identification (qualifier) and quantification (quantifier) of the analytes. A) MS spectrum of colistin obtained from the Scan mode of the MS instruments – the dominant peaks with *m*/*z* values of 390.7 and 386.0 represent parent ions of CST A and CST B, respectively. B) MS spectrum of CST A obtained from the Product ion mode of the MS instrument. The *m*/*z* values marked in red represent the identification and quantification ions. C) MS spectrum of CST B obtained from the Product ion mode of the MS instrument. The *m*/*z* values marked in red represent the identification and quantification ions. D) MS spectrum of polymyxin B obtained from the Scan mode of the MS instruments – the dominant peak with *m*/*z* value of 402.0 represents the parent ion of polymyxin B. E) MS spectrum of polymyxin B obtained from the Product ion mode of the MS instrument. The *m*/*z* values marked in red represent the identification and quantification ions.

Utilizing several QqQ operating modes, such as Scan, Selected Ion Monitoring (SIM), Product Ion, and Multiple Reaction Monitoring (MRM), in a sequential manner allowed for the optimization of the tandem mass spectrometry (MS/MS) step. Using these modes methodically allowed for the identification of precursor (parent) and product (daughter) ions, as well as the selection of precursor-product ion transitions for the unambiguous identification and determination of the analytes, as well as the setting of optimal values for parameters like fragmentor and collision energy. The precursor ions of CST A (*m*/*z* = 390.7), CST B (*m*/*z* = 386.0), and IS – polymyxin B (*m*/*z* = 402.0), were identified in Scan mode (Fig. 4). Based on the molecular weights of CST A (MW = 1169.5), CST B (MW = 1155.4) and polymyxin B (MW = 1203.5), all three analytes were triply charged preferentially under the selected conditions. These findings are consistent with previous papers using MS to detect CST [33]. The fragmentor voltage was optimized in SIM mode within the 20–200 V range. For CST A, CST B, and polymyxin B, the fragmentor voltage settings of 140 V and 100 V, respectively produced the best results in terms of the highest intensity of the precursor ions. A characteristic MS spectrum of the analytes appropriate for the selection of the suitable product ions was obtained by optimizing the collision energy (tested in the range of 5–20 eV) in the Product Ion mode. With nitrogen serving as the collision gas, the ideal collision energy for CST A, CST B, and polymyxin B was 20 eV and 15 eV, respectively.

Within the MS spectrum for every compound, two distinctive product ions were chosen: the qualifier (ion used for identity confirmation) and the quantifier (an ion with the highest signal intensity). To enhance the CST's quantification and qualification criteria, the MRM mode was employed finally. The following *m*/*z* ion transitions were applied in the MRM mode for CST A 390.7 → 101.1 (quantification transition), 390.7 → 211.6 (identity confirmation transition), CST B 386.0 → 101.0 (quantification transition) and 386.0 → 326.9 (identity confirmation transition), and polymyxin B 402.0 → 101.0 (quantification transition) and 402.0 → 378.4 (identity confirmation transition) (see Fig. 4).

The dwell time (the time spent acquiring a specific MRM transition during each cycle) is also an important parameter in CE-MS analysis because it can affect the S/N ratio and sensitivity. We investigated the effect of dwell time (in the range of 75–200 ms) on the CST signal intensity (Fig. S2 in Supporting Information). As the dwell time increased, an increase in the S/N and the peak area with a maximum at 150 ms was observed. Further increasing the dwell time decreased the S/N. Therefore, the dwell time of 150 ms was selected as the optimal one.

### 3.3 Optimization of plasma sample preparation

Effective pretreatment of biological samples is a crucial element of the whole analysis. A simple procedure based on plasma protein precipitation with organic solvent (ACN) was used here. It was demonstrated that a precipitation solution containing a small amount of HFo could increase the final analytical signal of CST. The addition of 1 % HFo to the precipitation solution resulted in a 2-fold higher analytical signal compared to pure ACN, but the analyses were not repeatable, and the current was not stable during the CE run. Using 0.1 % HFo in the precipitation solution offered a stable current and provided a 50 % increase in the analytical signal compared to ACN alone. Therefore, this solution was used in further experiments.

### 3.4 Method validation

The developed and optimized CE-MS method for the determination of CST was validated according to the recommendations of the Food and Drug Administration (FDA) guidelines [61]. Validation parameters such as selectivity, linearity range, accuracy, precision, the limit of detection (LOD), the lower limit of quantitation (LLOQ), recovery, and robustness were investigated. The evaluated operation and validation parameters are summarized in Table 2, Table 3, Table 4 and discussed below.

**Table 2:** Operation and calibration parameters of the CE-MS/MS method for determination of CST A and CST B in model water and plasma samples.

Parameter	CST A		CST B	
	Water	Plasma	Water	Plasma
t_m_, n = 6	11.98	12.75	11.94	12.68
RSD_tm_ (%), n = 6	0.02	0.09	0.08	0.12
RSD_area_ (%), n = 6	8.85	5.48	5.32	9.42
a (counts)	0.0271	−0.0015	0.1916	0.1915
SD_a_	0.0154	0.0355	0.0246	0.0315
b (counts.μg^−1^.mL)	0.9604	1.4831	0.9059	1.3168
SD_b_	0.0626	0.1382	0.1510	0.2001
r^2^	0.9962	0.9921	0.9905	0.9932
LOD (μg.mL^−1^)	0.0186	0.0186	0.0314	0.0314
LLOQ (μg.mL^−1^)	0.0372	0.0372	0.0628	0.0628
N	244,083	218,767	439,642	283,056

t_m_ – average migration time calculated at the first calibrator level; RSD_tm_ – relative standard deviation of normalized t_m_; RSD_area_ – relative standard deviation of peak area; a – intercept of the calibration curve; SD_a_ – standard deviation of intercept a; b – slope of the calibration curve; SD_b_ – standard deviation of slope b; r^2^ – coefficient od determination. LOD and LLOQ were calculated according to the signal-to-noise ratio (S/N), where LOD = 3*S/N, and LLOQ = 5*S/N. Separation efficiency (N) represented by the number of theoretical plates was calculated according to the following equation: N = 16*(t_m_/w)^2^, where t_m_ is migration time, and w is the peak width.

**Table 3:** Accuracy, precision and recovery of the CE-MS/MS method for CST A and CST B in plasma QC samples.

		QC low		QC medium		QC high	
		CST A	CST B	CST A	CST B	CST A	CST B
	Nominal (μg.mL^−1^)	0.28	0.47	2.79	4.71	6.51	10.99
Intra-day, n = 3	Found (μg.mL^−1^)	0.28	0.48	2.76	4.73	6.57	11.13
	RSD (%)	2.7	2.1	1.3	2.0	3.5	1.1
	RE (%	1.1	2.5	−1.1	0.6	1.0	1.3
Inter-day, n = 15	Found (μg.mL^−1^)	0.28	0.47	2.76	4.75	6.64	10.69
	RSD (%)	3.6	3.6	2.4	3.1	2.9	3.6
	RE (%)	0.8	0.6	−1.0	0.9	1.9	−2.7
Recovery (%), n = 3		92.5	99.5	95.8	97.3	96.7	98.0

**Table 4:** Stability testing of CST A and CST B in plasma QC samples.

		Autosampler stability, n = 5					Freeze-to-thaw stability, n = 5					
	QC low		QC medium		QC high		QC low		QC medium		QC high	
	CST A	CST B	CST A	CST B	CST A	CST B	CST A	CST B	CST A	CST B	CST A	CST B
Nominal (μg.mL^−1^)	0.28	0.47	2.79	4.71	6.51	10.99	0.28	0.47	2.79	4.71	6.51	10.99
Found (μg.mL^−1^)	0.29	0.46	2.94	4.66	6.76	10.78	0.29	0.49	2.81	4.67	6.54	10.82
RE (%)	−1.0	−1.4	3.3	−4.4	2.3	−0.3	2.7	3.0	1.7	−1.7	−1.6	1.7

RE (%) calculated as relative error in comparison to fresh sample.

The selectivity evaluation was based on measurements of blank plasma samples, zero calibrator plasma samples containing only IS, and plasma samples at the first calibration level. For these experiments, individual plasma samples obtained from six healthy volunteers were investigated. The obtained results clearly demonstrated that no matrix interferents were detected in the migration time of CST A, CST B, or 10.13039/100015147IS (illustrative records obtained during the investigation of a plasma sample are shown in Fig. S3 in Supporting Information).

The calibration curve was made from six calibration standards in aqueous and pooled plasma samples. The linear range of the method was for both matrices 0.19–7.44 μg mL^−1^ for CST A sulfate and 0.31–12.45 μg mL^−1^ for CST B sulfate. The calibration line parameters were calculated using Microsoft Excel 2007 (Microsoft Corporation, Redmond, WA). The appropriate linearity of the method was demonstrated by the coefficients of determination (r^2^) higher than 0.99 (Table 2). Moreover, the linearity of the method for both analytes was confirmed using the ANOVA test.

**Fig. 5:**
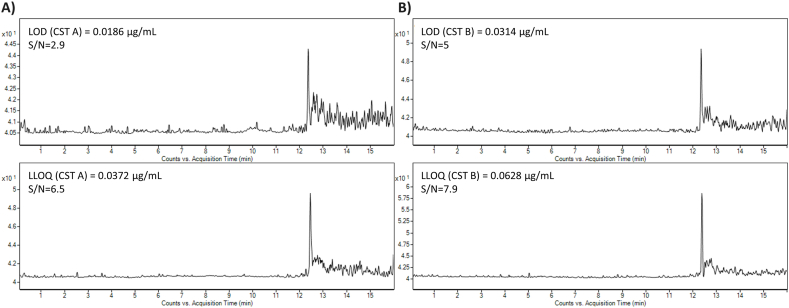
Illustrative MRM profiles of CST A (A) and CST B (B) obtained at the LOD (A, B upper traces) and LLOQ (A, B lower traces) concentrations levels. The profiles were obtained with the use of the quantitative MRM *m*/*z* transitions of CST A (390.7 → 101.1) and CST B (386.0 → 101.0). The samples were injected hydrodynamically using a pressure of 50 mbar for 20 s. The applied voltage was 25 kV and 50 mM HFo was used as BGE. For further details see section 2.2.

The LOD and LLOQ values were ascertained experimentally, the method based on S/N ratio was applied. LOD values (S/N = 3) for plasma and model water matrix were the same – i.e., 0.0186 μg mL^−1^ (CST A) and 0.0314 μg mL^−1^ (CST B). The LLOQ values (S/N = 5) for plasma and model water matrix were 0.0372 μg mL^−1^ (CST A) and 0.0628 μg mL^−1^ (CST B) (Fig. 5). In a pharmacokinetic study of pediatric patients, it was demonstrated that the mean steady-state concentration of CST in plasma (sum of CST A and CST B) ranged from 1.11 to 8.47 μg mL^−1^ (median, 2.92 μg mL^−1^) [62]. Moreover, the “Prato Polymyxin Consensus” also suggests as a reasonable target for CST (sum of CST A and CST B) an average steady-state concentration of 2 μg mL^−1^ [20]. The LLOQ values obtained by our CE-MS approach are more than appropriate for the required use.

The precision and accuracy of the developed CE-MS method were investigated using a series of QC samples prepared at three concentration levels – i.e. 0.28 and 0.47 μg mL^−1^ (QC low), 2.79 and 4.71 μg mL^−1^ (QC medium), and 6.51 and 10.99 μg mL^−1^ (QC high) for CST A and B, respectively (Table 3). Precision was examined in terms of repeatability within and between days. By testing the QC samples three times in a single day, the intraday precision was ascertained. Five days of repeated sample analysis, three replicates per day,were used to evaluate the interday precision. For CST A, the intraday accuracy (% RSD) varied between 1.3 and 3.5 %, while for CST B, it was between 1.1 and 2.1 %.The corresponding accuracy was expressed as % relative error – %RE, and calculated according to the following equation %RE = [(mean of measured concentration – nominal concentration)/nominal concentration]*100 %. The %RE varied from −1.1 to 1.1 % for CST A and from 0.6 to 2.5 % for CST B. The accuracy for CST A and CST B in the interday experiments ranged from 2.4 to 3.6 % and from 3.1 to 3.6 %, respectively. For CST A and CST B, the accuracy (% RE) ranged from −1.0 to 1.9 % and from −2.7 to 0.9 %, respectively. The FDA's requirements for accuracy and precision were satisfied, therefore the CE-MS method can reliably quantify CST A and CST B.

As a ratio of the analytical signals (normalized peak area of CST A or CST B) obtained from the analysis of plasma samples spiked with CST at three concentration levels (low, medium, and high) prior to extraction and comparative plasma samples spiked with CST following extraction, the analytical recovery of the method was assessed. The recoveries of CST A were 93 % (low QC), 96 % (medium QC), and 98 % (high QC). The recoveries of CST B were 99 % (low QC), 97 % (medium QC), and 98 % (high QC). The results indicated appropriate repeatability and acceptable recovery of CST from plasma samples.

During the validation process, two types of stability were assessed: freeze and thaw stability (three cycles of freezing and thawing at room temperature) and short-term stability (sample kept in an autosampler for 24 h at laboratory temperature). In order to compare the findings from the measurements of QC samples stored according to the guidelines with those of newly prepared QC samples, stability experiments were conducted. The findings in Table 4 unambiguously show that CST A and CST B in the plasma QC samples have satisfactory short-term and freeze-thaw stability. The FDA acceptance standard (±15 %) was satisfied by the differences between the nominal and found concentrations, as indicated by the relative error values.

Robustness is the ability of an analytical method to remain unaffected by small variations in the method parameters. Here, the effect of the change in BGE ±1 mM concentration was investigated using QC plasma samples. The %RE of the measured concentration achieved by comparing the QC samples measured at the changed BGE concentration with the QC samples measured at the original BGE concentration (50 mM HFo) was lower than 5 % in both cases. It is clearly shown that the method offers appropriate robustness.

The effect of carryover on the concentration accuracy of the studied samples was assessed by measuring the highest point of the plasma calibration curve of the CST reference standard followed by measuring the blank water sample. No peaks in the migration time of CST A or CST B in the blank water sample were observed (Fig. S4 in Supporting Information).

### 3.5 Method application

**Fig. 6:**
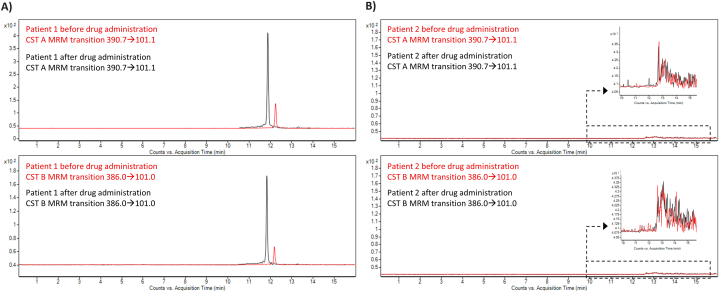
CE-MS/MS determination of CST in patient samples. A) Patient 1 is under CST treatment. Upper trace – MRM profiles obtained for CST A (*m*/*z* transition 390.7 → 101.1). Lower trace: MRM profiles obtained for CST B (*m*/*z* transition 386.0 → 101.0). B) Patient 2 is under the ATB treatment without CST. Upper trace – MRM profiles obtained for CST A (*m*/*z* transition 390.7 → 101.1). Lower trace: MRM profiles obtained for CST B (*m*/*z* transition 386.0 → 101.0). The samples were injected hydrodynamically using a pressure of 50 mbar for 20 s. The applied voltage was 25 kV and 50 mM HFo was used as BGE. For further details see section 2.2.

The application potential of the validated CE-MS method was tested on the plasma samples obtained from the Clinic of Hematology and Transfusiology of St Cyril and Methodius Hospital in Bratislava. Samples were collected from two critically ill patients treated with multiple intravenous antibiotics and antifungals. The samples were collected before and 30 min after the intravenous (i.v.) administration of the drugs. Patient 1 was treated with meropenem, trimethoprim/sulphamethoxazol, and CST. CST administered as CMS at the dose of 9,000,000 international units (IU) divided into three infusions was used to treat *Pseudomonas* infection. Patient 2 was treated with meropenem, vancomycin, trimethoprim/sulphamethoxazol, and fluconazole. Fig. 6 provides an overview of representative records derived from the analysis of the plasma samples. The total sum of the CST A and CST B bases was used to calculate the pure base, which represents the concentration of CST found in the clinical samples. The measured plasma CST concentration of patient 1 before drug administration was 0.82 ± 0.02 μg mL^−1^ (RSD = 5.3 %, n = 3). Plasma CST concentration 30 min after the multidrug administration was 4.58 ± 0.17 μg mL^−1^ (RSD = 7.5 %, n = 3) in patient 1. No analytical signal for CST was observed in plasma samples from patient 2 before and after administration of ATBs.

As previously stated, CST is a heterogeneous substance and, as such, differs not only between producers but also across batches produced by the same manufacturer. Therefore, the CST A/CST B ratio was also investigated in the case of clinical samples. In our work, the CST A/CST B ratio was 37.2/62.8 % in the samples of the reference standard. In clinical plasma samples, a reverse percentage representation of the CST forms was detected. Here, CST A was present at 76.4 % and CST B at 23.6 % level.

### 3.6 Method evaluation

#### 3.6.1 Comparison with LC approaches

The newly developed CE-MS approach for the bioanalysis of colistin in plasma was compared with the LC methods. An overview of the methods and their most important working parameters is summarized in Table 5.

**Table 5:** Overview of the LC and CE approaches used for determination of CST in biological samples.

Method	Matrix	Sample preparation	t (min)	LLOQ (ng.mL^−1^)^a^	Consumables	Ref.
LC-FLD	Serum, urine, sputum	Protein precipitation, derivatization	∼15	28.3	€€	[26]
LC-FLD	Plasma	Protein precipitation, SPE, derivatization	∼5	100	€€	[27]
LC-FLD	Plasma, urine, sputum	Precipitation, derivatization	∼18	n.a.	€€	[28]
LC-MS/MS	Plasma, culture medium	Protein precipitation	10	19.4 (CST A) 10.5 (CST B)	€€€	[29]
LC-MS/MS	Plasma, urine, perfusate	Protein precipitation, SPE	2	28 (CST A) 16 (CST B)	€€€	[30]
LC-MS/MS	Plasma, urine	SPE	3.8	24 (CST A) 15 (CST B)	€€€	[31]
LC-MS/MS	Plasma	SPE	2	n.a.	€€€	[32]
UHPLC-MS/MS	Plasma	SPE	2.5	20 (CST A) 30 (CST B)	€€€	[33]
UHPLC-MS/MS	Plasma	Protein precipitation	10	130 (CST A)^b^ 270 (CST B) b	€€€	[34]
LC-FLD	Plasma	Protein precipitation, SPE, derivatization	22	300*^2^	€€	[35]
LC-MS/MS	Plasma, ultrafiltrate	SPE	11	152 (CST A) 306 (CST B)	€€€	[36]
HILIC-MS/MS	Plasma	SPE	7	137 (CST A)^b^ 63 (CST B)^b^	€€€	[37]
UHPLC-MS/MS	Plasma, urine	Protein precipitation, SPE	3.5	13 (CST A) 25.1 (CST B)	€€€	[38]
UHPLC-MS/MS	Plasma, urine	Protein precipitation, SPE	2.5	24 (CST A) 12 (CST B)	€€€	[39]
UHPLC-MS/MS	Plasma, dried blood spot	Protein precipitation, extraction	3.5	100 (CST A) 200 (CST B)	€€€	[40]
LC-MS/MS	Plasma	Protein precipitation, SPE	4	50 (CST A)^b^ 50 (CST B)^b^	€€€	[41]
LC-MS/MS	Rat plasma	Protein precipitation, SPE	3	500	€€€	[42]
HILIC-MS/MS	Plasma, bile	Protein precipitation, SPE	12	9 (CST A) 2 (CST B)	€€€	[43]
LC-MS/MS	Rat plasma	Protein precipitation	3.5	7.3	€€€	[44]
LC-MS/MS	Plasma	Protein precipitation, extraction	5	300 (CST A) 500 (CST B)	€€€	[45]
CZE-MS/MS	Plasma	Protein precipitation	∼15	37.2 (CST A) 62.8 (CST B)	€	This study

**Abbreviations:** FLD – fluorescence detector, HILIC – hydrophilic interaction liquid chromatography, LC – liquid chromatography, MS/MS – tandem mass spectrometry, n.a. – not mentioned, SPE – solid phase extraction, UHPLC – ultra high-performance liquid chromatography.

a LLOQ assessed in plasma samples.

b LOQ.

Methods based on LC in combination with fluorescence (FLD) and MS detection are the dominant ones. In the case of LC-FLD analyses, derivatization of the sample was necessary, which significantly improved the time, number of pre-analytical steps (manipulation with the sample), and consumables demanded for sample preparation. Typically, multistep sample preparation (protein precipitation, extraction, and derivatization) was required in such analytical approaches [[25], [26], [27],^34^]. The need for extensive sample preparation typically increases the total cost of the analysis.

LC-MS/MS approaches offered a higher level of selectivity and were also used for simultaneous analysis of other antibiotics – e.g., vancomycin, teicoplanin, daptomycin, β-lactams, etc. [34,44,45]. Similarly, as in the case of the LC-FLD approaches, most of the LC-MS/MS methods were associated with the use of SPE sample pretreatment strategies applied separately [[31], [32], [33],^36^,^37^], or in combination with protein precipitation [30,[38], [39], [40], [41], [42], [43]]. Only a few LC-MS/MS deal with the simple one-step protein precipitation procedure [29,34,44]. The time of analysis by LC methods varies from 2.5 to 20 min. LLOQ values were predicted in the range of 9–300 ng mL^−1^ for CST A and in the range of 2–500 ng mL^−1^ for CST B.

Our newly developed CZE-MS/MS approach was able to offer LLOQ levels for CST A and CST B comparable to those obtained by the LC methods. Typically, a longer time of analysis was demanded for the realization of the CZE-MS/MS experiments (approximately 15 min). However, in some cases, our newly developed approach was faster than LC methods [28,35]. The CZE-MS/MS represents a very attractive approach and its benefits can be seen especially in high separation efficiency (number of theoretical plates >200,000) and minimal sample consumption – here, 20 nL of the sample was injected into the CE system compared to tenths of μL injected into the LC systems. According to these facts, it can be stated that our CE method can serve as a comparative and/or alternative to the routine and well-established LC approaches. Moreover, the developed CE method is also attractive from an economical point of view. Acquisition, operation, and consumables costs are significantly lower compared to the LC methods.

#### 3.6.2 Greenness assessment

**Fig. 7:**
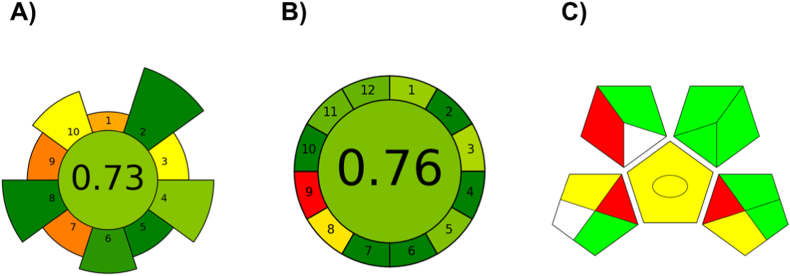
Greenness evaluation of the developed CE-MS method. Results obtained from the application of various greenness assessment metrics A) AGREEprep, B) AGREE, C) GAPI.

For the first time, the greenness assessment of a CE-MS approach with the potential to be applicable to real clinical practice was realized. The greenness of the developed and validated method was investigated using the Analytical Greenness Calculator (AGREE) [63] and the Green Analytical Procedure Index (GAPI) [64] metric tools. Moreover, the greenness of the sample preparation procedure was investigated using the AGREEprep tool [65]. The obtained results presented as pictograms are summarized in Fig. 7.

Firstly, the greenness of the sample preparation procedure was investigated with the use of the new metric tool – AGREEprep. This metric takes into the mind ten principles of green sample preparation during the evaluation procedure. Each of the inputs is transformed into a common scale in the 0–1 range. The estimated AGREEprep value of our developed method was 0.73 (Fig. 7A).

Further, AGREE is a complex greenness evaluation tool that takes into the mind all twelve principles of green analytical chemistry. The AGREE pictogram is a circle divided into twelve parts. Each part represents one separate green principle. Similarly, as in the case of AGREEprep, each of the inputs is transformed into a common scale in the 0–1 range. The estimated AGREE value of the developed CE-MS method was 0.76 (Fig. 7B).

Finally, the greenness assessment of the developed method was investigated with the use of GAPI. The results are summarized in a color scale pictogram composed of five pentagrams, which respond to sample preparation, reagents and solvents used, and instrumentation. Green color refers to safer impacts to nature, yellow represents problematic impacts, and red refers to hazardous impacts to nature that should be avoided. The GAPI pictogram of the proposed method was composed of 7 green, 3 yellow, 2 white, and 3 red fields (Fig. 7C).

The obtained results have clearly proved that the sample preparation procedure and the newly developed CE-MS method for the determination of CST in the clinical environment can be characterized as ecofriendly.

## 4 Conclusion

Here, we describe for the first time a novel CE-MS method suitable for the determination of colistin in human plasma samples. The developed method has several advantages such as simplicity, high separation efficiency, high level of selectivity, no excessive sample pretreatment, minimal sample consumption, and appropriate sensitivity. Furthermore, the developed CE-MS approach can be considered as green, as proved by the AGREE and GAPI greenness assessment tools. Similarly, the greenness of the sample preparation procedure was also confirmed by the AGREEprep metrics tool. To the best of our knowledge, our method is the first to use the CE-MS approach for determining CST in a clinical setting. Furthermore, the results demonstrated its reliability and potential to be incorporated into standard TDM for patients in critical condition. A cost-effective analytical approach like this will improve colistin use while reducing toxicity and adverse effects.

## Ethical statement

All subjects gave their informed consent for inclusion before they participated in the study. The study was conducted in accordance with the Declaration of Helsinki, and the protocol was approved by the Medical Ethical Committee of St Cyril and Methodius Hospital, Bratislava, Slovakia (Protocol EK1/10/2022).

## Data availability statement

Data associated with this study has not been deposited into a publicly available repository. Data will be made available on request.

## CRediT authorship contribution statement

**Ivana Cizmarova:** Writing – original draft, Validation, Methodology, Investigation, Data curation, Conceptualization. **Vojtech Parrak:** Investigation, Data curation, Conceptualization. **Peter Secnik jr:** Formal analysis, Data curation. **Peter Secnik:** Formal analysis, Data curation. **Ladislav Sopko:** Formal analysis, Data curation. **Kristina Nemergutova:** Formal analysis, Data curation. **Andrej Kovac:** Writing – review & editing, Visualization, Validation, Conceptualization. **Peter Mikus:** Writing – review & editing, Supervision. **Juraj Piestansky:** Writing – review & editing, Validation, Methodology, Investigation, Data curation, Conceptualization.

## Declaration of competing interest

The authors declare that they have no known competing financial interests or personal relationships that could have appeared to influence the work reported in this paper.
