# Supplementary material for: A simple and green capillary electrophoresis-mass spectrometry method for therapeutic drug monitoring of colistin in clinical plasma samples
Source: Heliyon. 2023 Nov 30;9(12):e23111. doi: 10.1016/j.heliyon.2023.e23111 (PMC10703858; doi:10.1016/j.heliyon.2023.e23111)
Supplement: Multimedia component 1 [file mmc1.docx]

**Supporting Information**

**A simple and green capillary electrophoresis-mass spectrometry method for therapeutic drug monitoring of colistin in clinical plasma samples**

Ivana Cizmarova^a,b^, Vojtech Parrak^c^, Peter Secnik jr^d^, Peter Secnik^d^, Ladislav Sopko^e^, Kristina Nemergutova^e^, Andrej Kovac^c^, Peter Mikus^a,b^, Juraj Piestansky^b,c,f*^

^a^Department of Pharmaceutical Analysis and Nuclear Pharmacy, Faculty of Pharmacy, Comenius University in Bratislava, Odbojarov 10, SK-832 32 Bratislava, Slovak Republic

^b^Toxicological and Antidoping Center, Faculty of Pharmacy, Comenius University in Bratislava, Odbojarov 10, SK-832 32 Bratislava, Slovak Republic

^c^Institute of Neuroimmunology, Slovak Academy of Sciences, Dubravska cesta 9, 84510 Bratislava, Slovakia

^d^SK-Lab s.r.o., Partizanska 15, SK-984 01, Lucenec, Slovak Republic

^e^Clinic of Hematology and Transfusiology, St Cyril and Methodius Hospital, Antolska 11, SK-851 07, Bratislava, Slovak Republic

^f^Department of Galenic Pharmacy, Faculty of Pharmacy, Comenius University in Bratislava, Odbojarov 10, SK-832 32 Bratislava, Slovak Republic

**Running title:** CE-MS method for TDM of colistin in clinical samples

*Corresponding Author:

Assoc. prof. PharmDr. Juraj Piešťanský, PhD.

Department of Galenic Pharmacy

Faculty of Pharmacy, Comenius University,

Odbojarov 10, SK-832 32 Bratislava, Slovak Republic

Phone: **421-2-50 117 250

E-mail: [piestansky@fpharm.uniba.sk](mailto:piestansky@fpharm.uniba.sk)

**Figure S1:** The effect of sheath liquid composition on the CST peak intensity.

**Figure S2:** The effect of dwell time on the CST peak intensity and peak area.

**Figure S3:** Specificity and selectivity proof based on comparison of plasma blank sample (green), zero (red) and first (black) calibrator.

**Figure S4:** Confirmation of no carry-over effect by comparing the highest calibrator level (black) with the blank water sample (red) measurement following right after it.


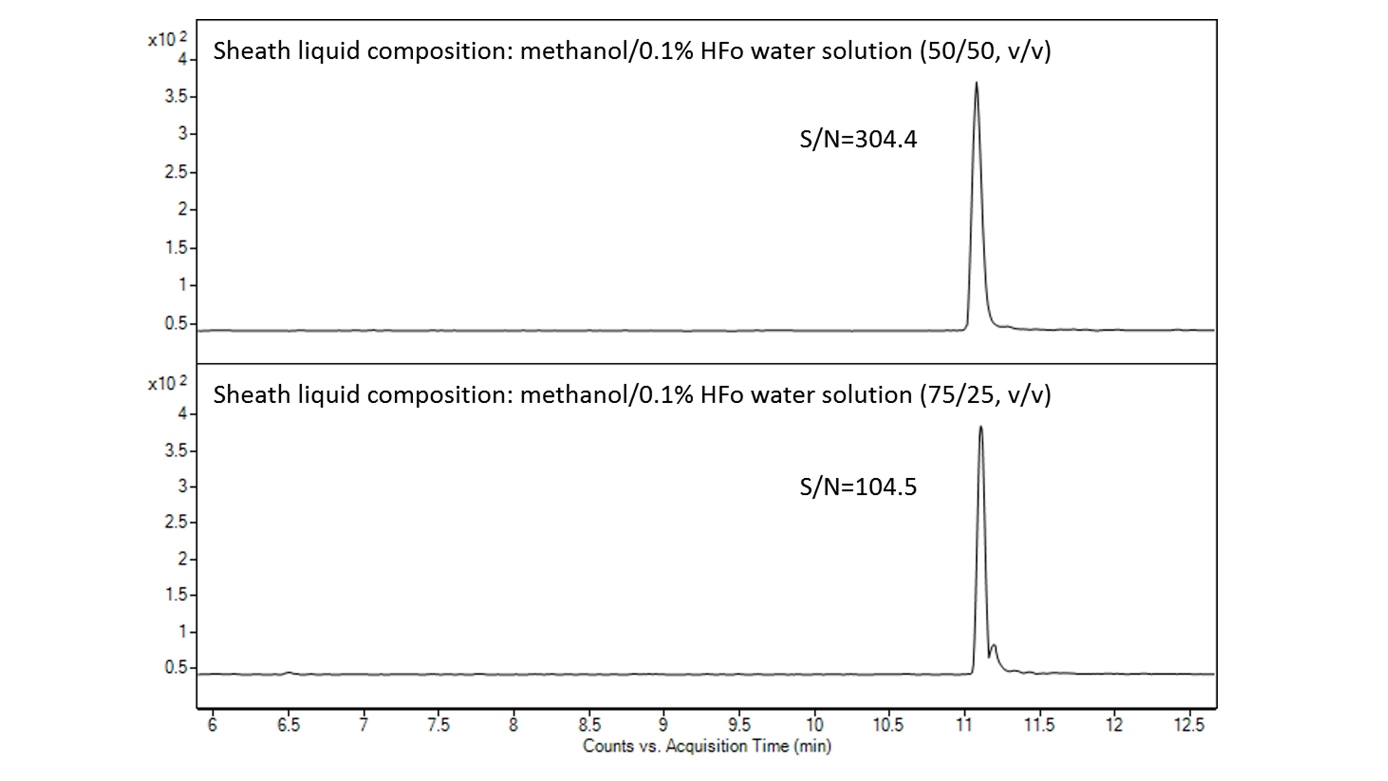


**Figure S1:** The effect of sheath liquid composition on the CST peak intensity. Upper trace: Multiple reaction monitoring (MRM) profile of CST B obtained with the use of sheath liquid composed of mixture methanol/0.1% HFo in water (50/50, v/v). Lower trace: Multiple reaction monitoring (MRM) profile of CST B obtained with the use of sheath liquid composed of mixture methanol/0.1% HFo in water (75/25, v/v). The presented CZE profiles were obtained with the use of the quantitative MRM m/z transition of CST B, i.e. 386.0 →101.0. The concentration of injected CST sulfate salt was 10 μg.mL^-1^, what represents concentration 6.28 μg.mL^-1^ of CST B sulphate salt. The sample was injected hydrodynamically using a pressure of 50 mbar for 20 seconds. The applied voltage was 25 kV and 50 mM HFo was used as BGE. For further details see section 2.2.


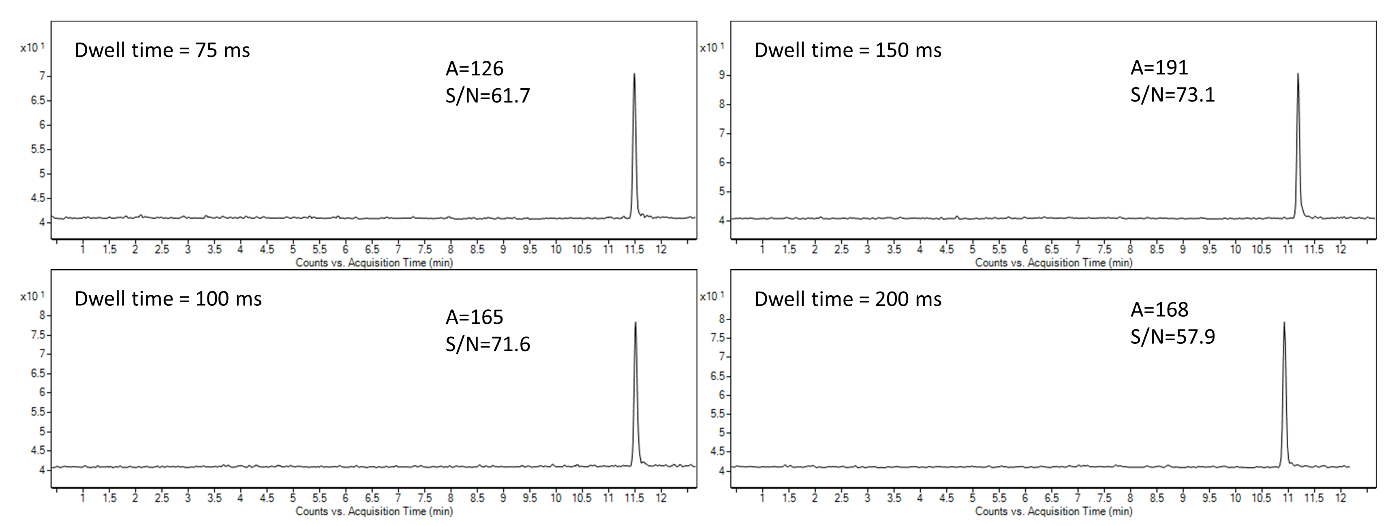


**Figure S2:** The effect of dwell time on the CST peak intensity and peak area. The presented CZE profiles were obtained with the use of the quantitative MRM m/z transition of CST B, i.e. 386.0 →101.0. The concentration of injected CST sulfate salt was 10 μg.mL^-1^, what represents concentration 6.28 μg.mL^-1^ of CST B sulfate salt. The sample was injected hydrodynamically using a pressure of 50 mbar for 20 seconds. The applied voltage was 25 kV and 50 mM HFo was used as BGE. For further details see section 2.2.


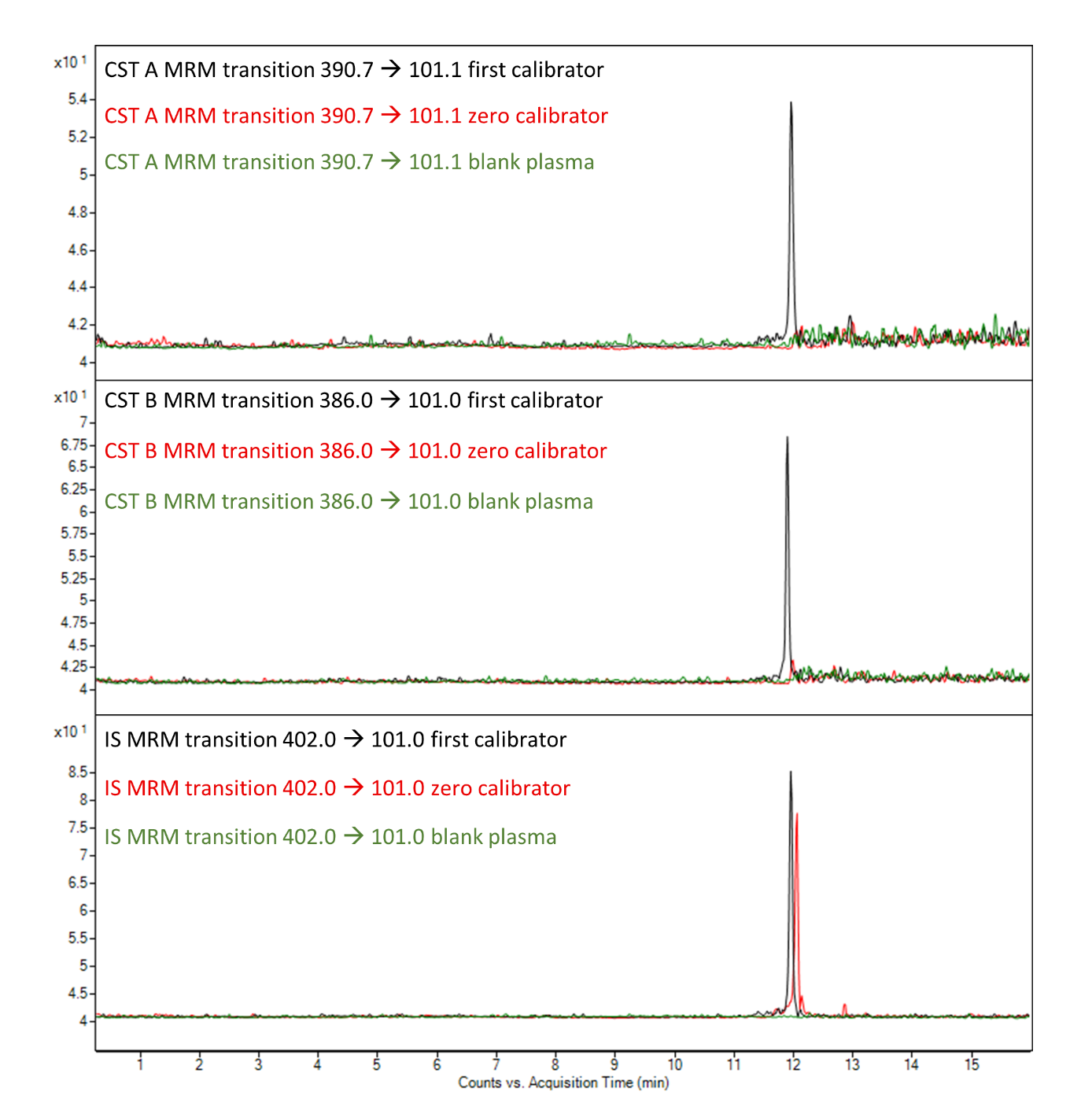


**Figure S3:** Specificity and selectivity proof based on comparison of plasma blank sample (green), zero (red) and first (black) calibrator. Upper trace: Multiple reaction monitoring (MRM) of CST A. Middle trace: Multiple reaction monitoring (MRM) of CST B. Lower trace: Multiple reaction monitoring (MRM) of polymyxin. The samples were injected hydrodynamically using a pressure of 50 mbar for 20 seconds. The applied voltage was 25 kV and 50 mM HFo was used as BGE. For more details see section 2.2.


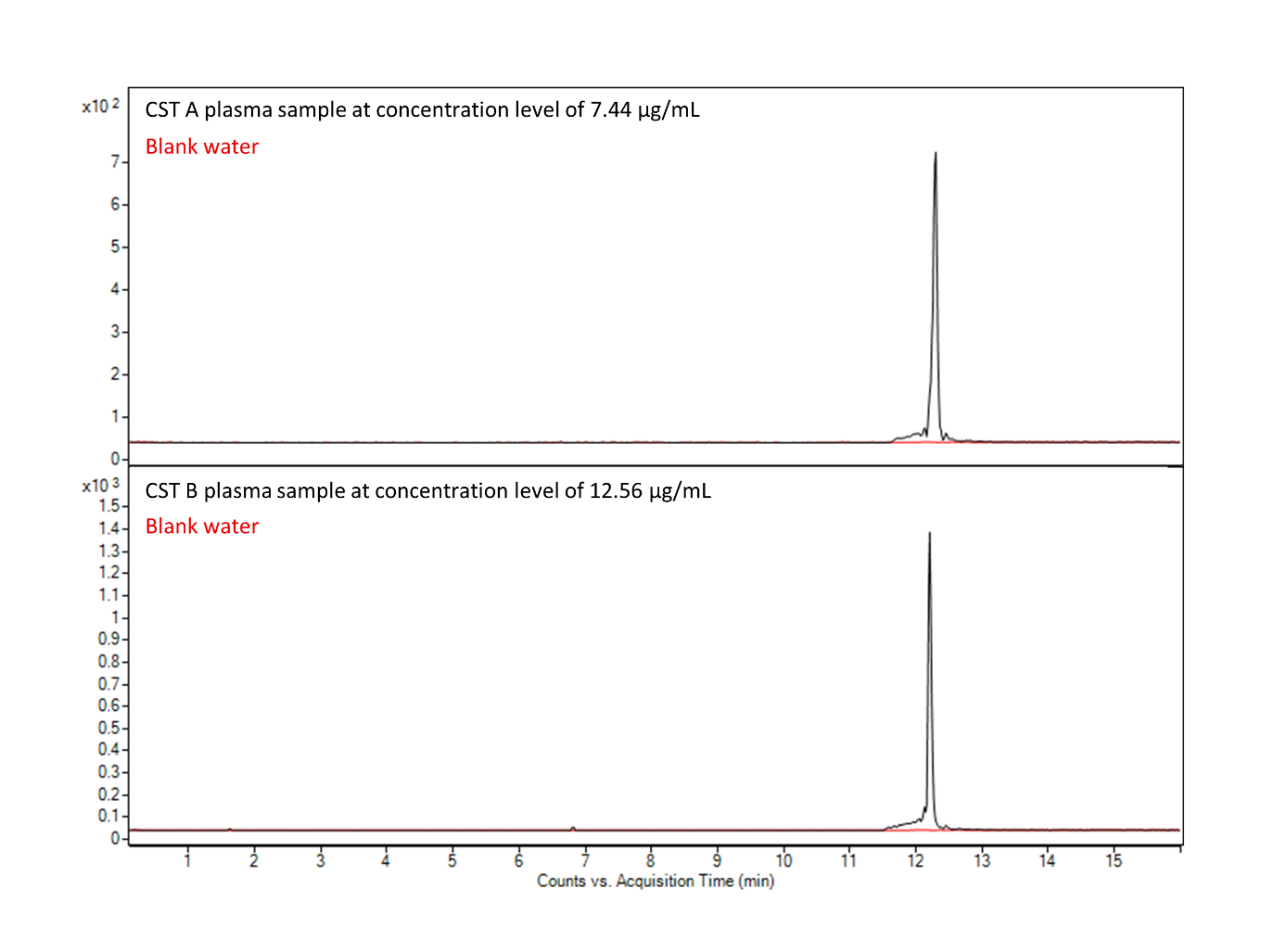


**Figure S4:** Confirmation of no carry-over effect by comparing the highest calibrator level (black) with the blank water sample (red) measurement following right after it. Upper trace: MRM profiles obtained for CST A (m/z transition 390.7 → 101.1). Lower trace: MRM profiles obtained for CST B (m/z transition 386.0 →101.0). The samples were injected hydrodynamically using a pressure of 50 mbar for 20 seconds. The applied voltage was 25 kV and 50 mM HFo was used as BGE. For further details see section 2.2.
